# Influencing factors of pressure pain hypersensitivity of the upper trapezius in food service workers with nonspecific neck/shoulder myofascial pain: A cross-sectional study

**DOI:** 10.1097/MD.0000000000029696

**Published:** 2022-08-05

**Authors:** Ui-Jae Hwang, Oh-Yun Kwon

**Affiliations:** a Department of Physical Therapy, Graduate School, Yonsei University, Wonju, Kangwon-Do, South Korea; b Department of Physical Therapy, College of Health Science, Laboratory of Kinetic Ergocise Based on Movement Analysis, Yonsei University, Wonju, Kangwon-Do, South Korea.

**Keywords:** influencing factors, myofascial pain, pressure pain hypersensitivity, upper trapezius

## Abstract

It is unclear which factors contribute to the developing pressure pain hypersensitivity of the upper trapezius, a type of neurophysiological hyperexcitability. The present study investigated the relationship between physical and psychological factors and pressure pain hypersensitivity of the upper trapezius for each sex. In total, 154 individuals with neck/shoulder myofascial pain participated, among 372 food service workers. Participants completed a questionnaire (Beck Depression Inventory, and Borg Rating of Perceived Exertion scale) and were photographed to measure posture. Pressure pain sensitivity, 2 range of motions (cervical lateral bending and rotation), and 4 muscle strengths (serratus anterior, lower trapezius [LT], biceps, and glenohumeral external rotator) were measured by a pressure algometer, iPhone application, and handheld dynamometer, respectively. For each sex, forward multivariate logistic regression was used to test our a priori hypothesis among selected variables that a combination of psychosocial and physical factors contributed to the risk for pressure pain hypersensitivity. In multivariate analyses, LT strength (odds ratio = 0.94, 95% confidence interval = 0.91–0.97, *P* = .001) was the only significant influencing factor for pressure pain hypersensitivity in men. Dominant painful ipsilateral cervical rotation range of motion (odds ratio = 0.96, 95% confidence interval = 0.92–0.99, *P* = .037) was the only influencing factor for pressure pain hypersensitivity in women. LT strength and dominant painful ipsilateral cervical rotation range of motion could serve as guidelines for preventing and managing pressure pain hypersensitivity of the upper trapezius in food service workers with nonspecific neck/shoulder myofascial pain.

## 1. Introduction

Because of the high strain related to serving, preparing raw materials, washing dishes, and cooking, food service workers (FWs), such as cooks and restaurant employees, are at high risk for musculoskeletal pain.^[1–3]^ A high prevalence of musculoskeletal pain in FWs has been reported. Among 905 individuals in 2 previous studies, the neck (54.3%) and shoulders (57.9%) were more involved than other body regions (22.3%–52.75%).^[4,5]^ A Norwegian study found that 80% of hotel FWs reported lifelong musculoskeletal pain, including 42.4% with neck/shoulder pain.^[6]^

One of the most common causes of musculoskeletal pain is myofascial pain (MP).^[7]^ The origin of MP is located at myofascial trigger points, which are hyperirritable regions of tenderness in the taut bands of skeletal muscles^[8]^ that become painful when stimulated (e.g., via compression or other mechanical stimulations) and can contribute to the generation of pain, motor dysfunction, and autonomic responses.^[8–10]^ Among postural muscles, the upper trapezius (UT) muscle is most affected by MP.^[7,11,12]^

Tenderness over muscles is a common clinical finding in painful conditions of presumed muscular origin.^[13,14]^ Pressure pain sensitivity (PPS) is a quantitative sensory test for assessing pain sensitivity in deep tissues.^[15]^ This neurophysiological test may be suitable to measure PPS and tissue tenderness because these conditions are believed to reduce the test values.^[15]^

Although the pathophysiologic mechanism of MP has not been identified, it may involve central sensitization (hyperresponsiveness and hyperexcitability of the central nervous system).^[16,17]^ However, it is unclear which factors increase the risk of developing pressure pain hypersensitivity (PPH) in terms of neurophysiological hyperresponsiveness and hyperexcitability. Suggested factors include individual factors (e.g., sex),^[18]^ physical factors (e.g., posture),^[19]^ and psychosocial factors (e.g., depression and stress).^[20]^ Previous studies have investigated the influences of individual factors on PPS. Thus, there is a need to investigate the combined influences of multiple factors on the PPS.

Physical influencing factors are useful and potentially could be modified with interventions such as exercise.^[21]^ Conversely, individual characteristics (e.g., sex and age) cannot be modified. To determine a specific management approach for neck/shoulder pain, Donatelli^[22]^ proposed examination of the following aspects: cervical and shoulder posture, muscle length, rotator cuff muscle strength, and scapular rotator strength. Psychosocial factors and increased pain sensitivity are phenotypic domains related to the risk of developing chronic musculoskeletal pain.^[23]^ Increased PPS may be indicative of altered central pain mechanisms by psychosocial factors.^[24]^ Psychosocial influencing factors include pain catastrophizing, kinesiophobia, anxiety, depression, and stress associated with boring and monotonous tasks, low social support, and low job satisfaction.^[25,26]^ Psychological, biological, and social domains could explain differences in pain severity and perception between men and women with MP.^[18,27]^ Concerning sex differences in pain outcomes, PPS has demonstrated the greatest effect size^[28]^ and women are more sensitive to pressure pain than men.^[29]^ Because of sex differences in pain perception and PPS, as well as differences in food service tasks, the relative contributions of physical and psychological influencing factors to PPH should be identified for each sex.

Therefore, the present study investigated differences in physical and psychosocial factors between participants with and without PPH for each sex and the relationship between physical and psychological factors and PPH for each sex.

## 2. Methods

### 2.1. Participants

**Figure 1.**
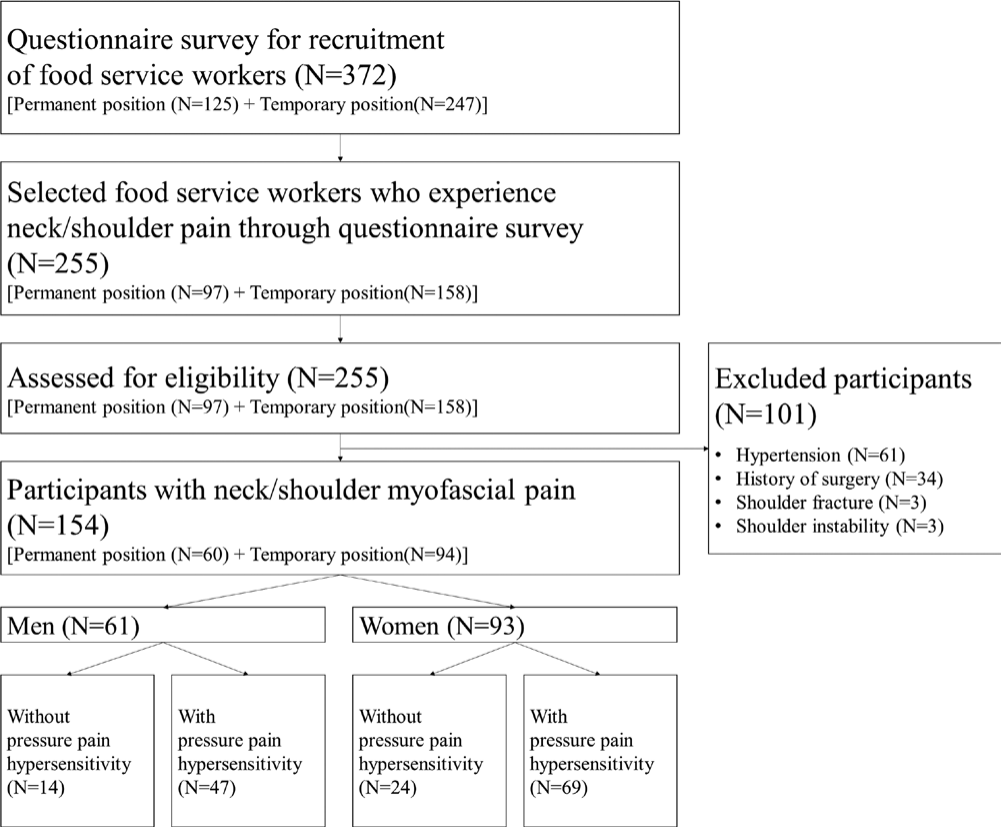
Flow diagram of participant selection.

Participants were recruited through a questionnaire to confirm their experience of neck/shoulder MP as FWs. In total, 154 individuals with neck/shoulder MP participated, from among 372 FWs in a theme park. A flowchart for recruitment of the participants is shown in Figure 1. Inclusion criteria were nontraumatic neck/shoulder pain, >6 months of work in food service, presence of neck/shoulder pain for ≥3 months, and visual analog scale score >30 mm. Exclusion criteria were shoulder fractures, a prior diagnosis of shoulder instability, a history of surgery in the shoulder, any systemic disease, untreated psychiatric condition, examination suggesting the presence of neurological diseases or internal diseases, hypertension (resting systolic blood pressure >150 mm Hg or diastolic blood pressure >90 mm Hg), and/or pregnancy. Participant characteristics are shown in Table 1. The experimental protocol was established according to the ethical guidelines of the Helsinki Declaration. The study protocol was approved by Institutional Review Board (certification number: #1041849–201603-BM-005–02). Before assessment, the investigator explained the entire experimental procedure and all participants voluntarily provided informed consent.

**Table 1:** Participant characteristics.

	Men (n = 61)	Women (n = 93)	Total (n = 154)
Age (yr)	32.05 (8.90)	26.15 (7.11)	28.49 (8.36)
Height (cm)	173.82 (5.40)	163.57 (6.06)	167.63 (7.67)
Weight (kg)	73.00 (8.44)	56.32 (7.75)	62.93 (11.45)
Body mass index (kg/m^2^)	24.18 (2.79)	20.93 (2.08)	22.22 (2.87)
Work duration (mo)	62.00 (70.16)	41.72 (59.49)	50.30 (64.80)
Pain dominant side	Rt: 30; Lt: 31	Rt: 46; Lt: 47	Rt: 76; Lt: 78
Pressure pain hypersensitivity	47/61	69/93	116/154
Visual analog scale	54.02 (23.09)	53.99 (19.12)	54.00 (20.71)

### 2.2. Outcome measurements

#### 2.2.1. Pressure pain sensitivity.

PPS was measured with the participant seated upright using a pressure algometer (Wagner Instruments, Inc., Greenwich, CT) with a 1-cm diameter rubber tip attached to a strain gauge that displayed values in kg/cm^2^. The tip was applied to the UT at a standardized location containing the midpoint between C7 and the acromion process, in the dominant painful side (intraclass correlation coefficient of interrater reliability: 0.91).^[30,31]^ PPS was defined as the lowest pressure at which the sensation of pressure turned to slight pain or discomfort.^[18,30,31]^ The mean value of 3 trials was calculated and used for the main analyses. A 1 minute resting period was allowed between each recording. Both the participant and examiner were blind to force readings during the assessment. A standard metronome was also used to control the rate of increase in pressure. Men with PPS <2.9 kg/cm^2^ in the UT and women with PPS <2.0 kg/cm^2^ in the UT were presumed to have PPH.^[32]^

#### 2.2.2. Psychological domain: depression.

The Beck Depression Inventory (BDI) is widely used to measure depression. Previous studies have supported the suitability of the BDI in assessing depression in patients with chronic musculoskeletal pain.^[33–35]^ The BDI consists of 21 items based on symptoms and attitudes.^[36]^ Statements were ranked to indicate the range of depression severity from neutral to maximal.

#### 2.2.3. Physical domains.

Exertion of work intensity was measured using the Borg rating of perceived exertion scale. Participants were asked to self-rate their exertion of work intensity on a scale between 6 and 20.^[37]^

**Figure 2.**
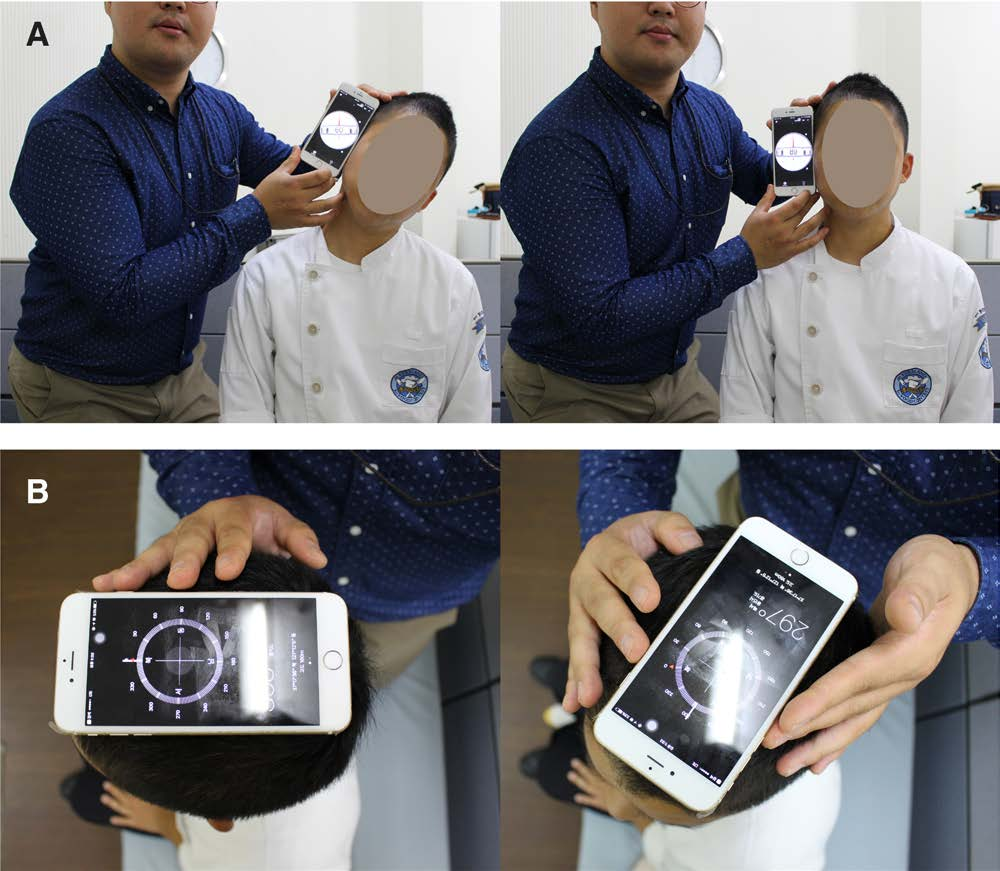
Measurement of range of motion: (A) cervical lateral bending and (B) rotation.

For cervical range of motion (ROM), the dominant painful contralateral cervical side-bending and dominant painful ipsilateral cervical rotation ROM were measured using an iPhone with Clinometer and Compass applications^[38,39]^ (Fig. 2). Using a belt strap, participants were blocked from performing trunk and shoulder movements during measurements of cervical lateral bending and rotation movement. The measurements of cervical ROM were made for the total range (i.e., difference between initial and final measures). The mean value of 3 trials was calculated and used for the main analyses.

**Figure 3.**
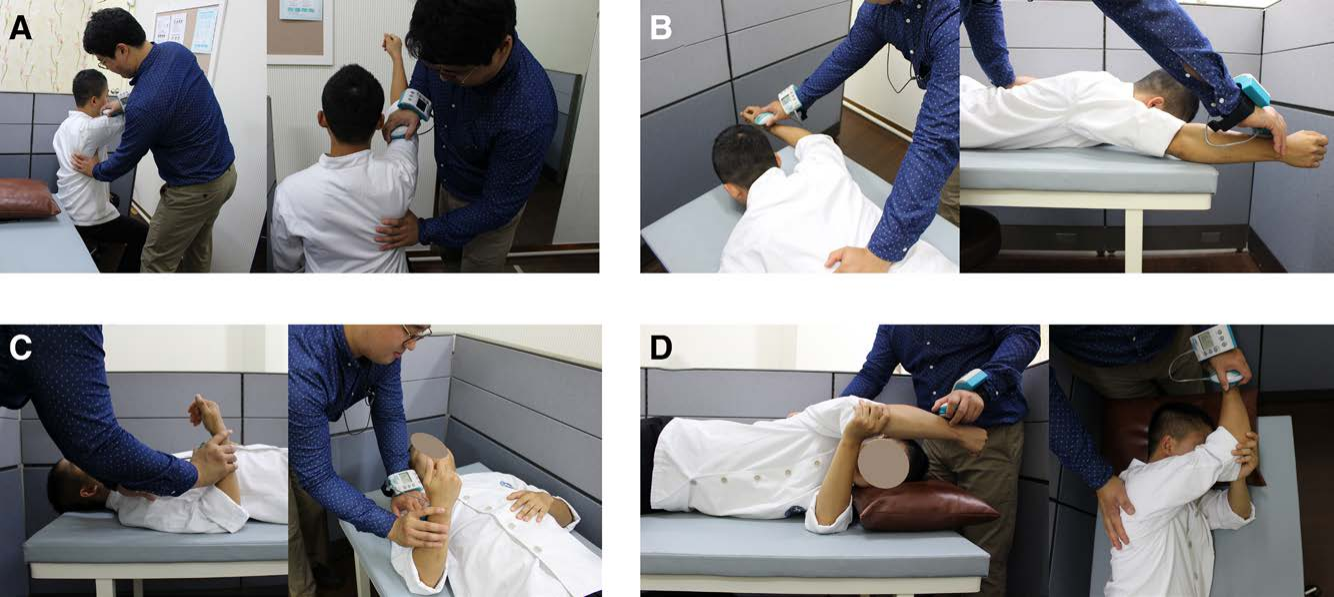
Measurement of muscle strength: (A) serratus anterior, (B) lower trapezius, (C) biceps and (D) glenohumeral external rotator.

For muscle strength, serratus anterior, lower trapezius (LT), biceps, and glenohumeral external rotator strengths were measured using a handheld dynamometer (JTECH Medical, Salt Lake City, UT) in the dominant painful side^[39]^ (Fig. 3). The unit of measurement was a Newton (N) generated by isometric contraction. Muscle strength values were normalized according to participant body weight. The mean value of 3 trials was calculated and used for analyses. Serratus anterior strength was measured in scapular protraction and the shoulder was flexed to 125°.^[40]^ Participants were instructed to hold the upper extremity position while the examiner provided a downward force with the handheld dynamometer immediately over the distal humerus. In the prone position, LT strength was measured with the upper extremity diagonally overhead, in line with the LT fibers.^[41]^ The handheld dynamometer was applied to the distal one-third of the participant’s radial forearm, and force toward the floor was applied by the examiner. Biceps strength was measured with participants in the sitting position with their elbow flexed to 90°.^[41]^ The handheld dynamometer force sensor was applied to the distal one-third of the participant’s forearm, and force toward the floor was applied by the examiner. Glenohumeral external rotator was measured in the side-lying position with the shoulder flexed and internally rotated to 90°, and the elbow flexed to 90°.^[39]^ Then the dynamometer was applied to the distal one-third of the participant’s radial forearm, and force toward the floor was applied by the examiner.

For posture analyses, forward head posture, rounded shoulder angle, shoulder slope angle, and scapular downward rotation ratio were measured using kinematic analyses of photographs using ImageJ software (National Institutes of Health, Bethesda, MD). Forward head posture was quantified by the craniovertebral angle (angle between the horizontal line passing through C7 and a line extending from the tragus of the ear to C7). The rounded shoulder angle was quantified using the angle (θ) between 2 lines (from a horizontal line in the medial roots of the scapula to the acromion, and from the root of the scapula to the acromion). The angle θ, composed of the 2 distances, is 1 apex of a right-angled triangle. Therefore, 90 – θ was defined as the rounded shoulder angle. Shoulder slope angle was quantified by the angle between 2 lines (a horizontal line with the acromion and a line between the spinous process of the seventh cervical vertebrae and acromion). Scapular downward rotation ratio was quantified by the ratio between 2 lines (a vertical line from the center to the root of the scapula, and a vertical line from the center to the scapular inferior angle).

### 2.3. Procedures

The present study was performed from March 2016 to November 2016. Participants were assessed at the work conditioning center in a theme park. Variables were measured in the following order: psychological and physical domains (posture, ROM, and strength). Participants were asked to complete a questionnaire (age, sex, BDI, and Borg rating of perceived exertion scale) and then were photographed to measure posture. PPS, 2 ROMs (cervical lateral bending and rotation), and 4 muscle strengths (serratus anterior, LT, biceps, and glenohumeral external rotator) were measured in that order. Two testers performed the measurements separately for interrater reliability. One tester screened for and assessed PPS, and the other tester measured the independents variables. The participants were blinded to the PPS data.

### 2.4. Statistical analyses

The Kolmogorov–Smirnov *Z* test was used to assess the assumption of distribution normality. Demographic characteristics are shown as means. Independent *t* tests were used to compare psychological and physical domains between participants with PPH and participants without PPH, and to identify significant variables for cutoff in each sex because of sex differences in demographic and clinical features. Variables with significant differences between participants according to PPH status in men and women were selected. For each sex, variables associated with PPH were selected from univariate analyses. Finally, for each sex, forward multivariate logistic regression was used to test our a priori hypothesis among selected variables that a combination of psychosocial and physical factors contributed to the PPH. The analyses were adjusted for previously established covariates of age and body mass index. Goodness-of-fit was calculated using the Hosmer–Lemeshow test. Statistical analyses were conducted using SPSS Statistics (version 18.0; IBM Corp., Armonk, NY) and the significance level was set at *P* < .05.

## 3. Results

### 3.1. Comparisons of psychological and physical domains in men according to PPH status

Table 2 shows comparisons of psychological and physical domains between men with PPH and men without PPH. There were no significant differences in BDI, Borg rating of perceived exertion scale, forward head posture, rounded shoulder angle, shoulder slope angle, or scapular downward rotation ratio between men with PPH and men without PPH. For cervical ROM, the dominant painful contralateral cervical side-bending (*P* = .02) and dominant painful ipsilateral cervical rotation ROM (*P* = .01) were significantly greater in men without PPH than in men with PPH. For muscle strengths, serratus anterior (*P* = .005), LT (*P* = .002), biceps (*P* < .001), and glenohumeral external rotator strength (*P* = .001) were significantly greater in men without PPH than in men with PPH.

**Table 2:** Comparisons of psychological and physical domains in men according to pressure pain hypersensitivity status (n = –PPH: 14, +PPH: 47).

Variables	Group	Mean (SD)	*P* value	95% CI
Pressure pain threshold (kg/cm^2^)	–PPH	3.03 (0.19)	.000*	1.03–1.31
	+PPH	1.86 (0.33)		
Beck Depression Inventory	–PPH	25.64 (3.25)	.988	–2.35 to 2.32
	+PPH	25.66 (5.17)		
Borg Rating of Perceived Exertion Scale	–PPH	13.50 (2.21)	.938	–1.47 to 1.36
	+PPH	13.55 (2.33)		
Cervical side-bending ROM (°)	–PPH	54.68 (8.07)	.007*	2.32–13.46
	+PPH	46.79 (11.46)		
Cervical rotation ROM (°)	–PPH	72.18 (7.01)	.058	–0.26 to 14.94
	+PPH	64.84 (13.63)		
Serratus anterior strength (normalize: %)	–PPH	242.06 (57.65)	.003*	22.66–99.43
	+PPH	181.01 (72.53)		
Lower trapezius strength (normalize: %)	–PPH	57.81 (24.95)	.000*	15.19–37.21
	+PPH	31.61 (15.58)		
Biceps strength (normalize: %)	–PPH	362.99 (73.88)	.000*	62.66–171.59
	+PPH	245.86 (93.32)		
GHER strength (normalize: %)	–PPH	76.32 (26.68)	.005*	8.26–41.38
	+PPH	51.50 (23.78)		
Rounded shoulder angle (°)	–PPH	36.56 (4.59)	.286	–4.50 to 1.39
	+PPH	38.11 (4.92)		
Froward head posture (°)	–PPH	58.77 (11.95)	.233	–3.00 to 11.57
	+PPH	54.49 (9.23)		
Shoulder slope angle (°)	–PPH	18.46 (2.09)	.939	–1.39 to 1.50
	+PPH	18.40 (2.96)		
Scapular downward rotation ratio	–PPH	0.90 (0.15)	.526	–0.07 to 0.13
	+PPH	0.87 (0.15)		

### 3.2. Comparisons of psychological and physical domains in women according to PPH status

Table 3 shows comparisons of psychological and physical domains between women with PPH and women without PPH. Most variables were not significantly different, but dominant painful ipsilateral cervical rotation ROM (*P* = .033) was significantly greater in women without PPH than in women with PPH.

**Table 3:** Comparisons of psychological and physical domains in women according to pressure pain hypersensitivity status (N = –PPH: 24, +PPH: 69).

Variables	Group	Mean (SD)	*P* value	95% CI
Pressure pain threshold (kg/cm^2^)	–PPH	2.36 (0.38)	.000*	0.64–0.90
	+PPH	1.60 (0.23)		
Beck Depression Inventory	–PPH	31.21 (5.91)	.666	–2.24 to 3.47
	+PPH	30.59 (6.09)		
Borg Rating of Perceived Exertion Scale	–PPH	13.29 (2.40)	.432	–1.59 to 0.69
	+PPH	13.74 (2.31)		
Cervical side-bending ROM (°)	–PPH	50.38 (7.42)	.328	–2.47 to 7.30
	+PPH	47.96 (11.20)		
Cervical rotation ROM (°)	–PPH	69.71 (9.52)	.019*	1.02–10.78
	+PPH	63.81 (12.13)		
Serratus anterior strength (normalize: %)	–PPH	176.17 (50.09)	.500	–16.20 to 32.71
	+PPH	167.91 (54.17)		
Lower trapezius strength (normalize: %)	–PPH	31.51 (11.39)	.349	–2.82 to 7.79
	+PPH	29.03 (10.00)		
Biceps strength (normalize: %)	–PPH	212.91 (58.75)	.088	–3.72 to 51.24
	+PPH	189.15 (52.51)		
GHER strength (normalize: %)	–PPH	49.29 (21.55)	.995	–8.00 to 8.05
	+PPH	49.26 (15.23)		
Rounded shoulder angle (°)	–PPH	37.62 (6.78)	.363	–1.74 to 4.63
	+PPH	36.17 (6.21)		
Froward head posture (°)	–PPH	51.87 (9.12)	.083	–8.82 to 0.56
	+PPH	56.00 (11.75)		
Shoulder slope angle (°)	–PPH	16.43 (3.95)	.065	–0.11 to 3.64
	+PPH	14.67 (3.81)		
Scapular downward rotation ratio	–PPH	0.87 (0.15)	.726	–0.08 to 0.06
	+PPH	0.88 (0.14)		

### 3.3. Multivariate prediction model for PPH of UT in men and women

By comparison of psychological and physical domains between participants with PPH and participants without PPH, the following variables were selected: dominant painful contralateral cervical side-bending, dominant painful ipsilateral cervical rotation ROM, serratus anterior, LT, biceps, and glenohumeral external rotator strength. The results of univariate analyses of predictors of PPH of UT in men and women are shown in Table 4 and Supplemental Digital Content 1, Supplemental Digital Content, http://links.lww.com/MD/G968. In univariate analyses, dominant painful contralateral cervical side-bending (odds ratio [OR] = 0.93, 95% confidence interval [CI] = 0.87–0.99), serratus anterior (OR = 0.99, 95% CI = 0.98–1.00), LT (OR = 0.94, 95% CI = 0.91–0.97), biceps (OR = 0.98, 95% CI = 0.96–0.99), and glenohumeral external rotator strength (OR = 0.96, 95% CI = 0.94–0.99) were significantly associated with PPH in men. Moreover, only dominant painful ipsilateral cervical rotation ROM (OR = 0.96, 95% CI = 0.92–1.00) was significantly associated with PPH in women.

**Table 4:** Predictors of pressure pain hypersensitivity using selected variables: results from univariate analysis.

Sex	Variables	*P* value	OR	95% CI
Male	Cervical side-bending ROM	.027*	0.93	0.87–0.99
	Cervical rotation ROM	.065	0.95	0.90–1.00
	Serratus anterior strength	.011*	0.99	0.98–1.00
	Lower trapezius strength	.001*	0.94	0.91–0.97
	Biceps strength	.002*	0.98	0.96–0.99
	GHER strength	.005*	0.96	0.94–0.99
Female	Cervical side-bending ROM	.325	0.98	0.93–1.02
	Cervical rotation ROM	.037*	0.96	0.92–1.02
	Serratus anterior strength	.510	1.00	0.99–1.01
	Lower trapezius strength	.313	0.98	0.94–1.02
	Biceps strength	.072	0.99	0.98–1.00
	GHER strength	.995	1.00	0.97–1.03

The results of adjusted multivariate analyses of predictors of PPH of UT in men and women are shown in Table 5 and Supplemental Digital Content 1, Supplemental Digital Content, http://links.lww.com/MD/G968. In the adjusted multivariate model, LT strength (OR = 0.94, 95% CI = 0.91–0.97, *P* = .001) was the only significant influencing factor for PPH of the UT in men. In addition, dominant painful ipsilateral cervical rotation ROM (OR = 0.96, 95% CI = 0.92–0.99, *P* = .037) was the only influencing factor for PPH of the UT in women. Goodness-of-fit statistics indicated that model fitting was appropriate for each sex-adjusted regression model (men: *P* = .290, women: *P* = .061).

**Table 5:** Predictors of pressure pain hypersensitivity using selected variables: results from adjusted multivariate analyses.

Sex	Variables	*P* value	OR	95% CI
Male	Lower trapezius strength	.001*	0.94	0.91–0.97
Female	Cervical rotation ROM	.037*	0.96	0.92–1.00

## 4. Discussion

PPS can result from impairments at multiple levels throughout the neuromuscular system.^[42]^ There is increasing evidence that changes in pain processing may enhance sensitivity to noxious stimuli among individuals with chronic pain, compared to pain-free controls.^[14,43]^ The present study investigated whether physical and psychological domains were related to PPH of the UT in each sex among FWs with nonspecific neck/shoulder MP, because biological differences have been suggested to cause sex differences in pain perception.^[44–46]^ LT strength and dominant painful ipsilateral cervical rotation ROM were characterized as influencing factors of PPH of the UT in male and female FWs with nonspecific neck/shoulder MP in adjusted multivariate analyses. Although our interpretations of causality are limited because of the cross-sectional study design, the LT strength and dominant painful ipsilateral cervical rotation ROM identified in the present study could be useful for establishing guidelines for the prevention and management of PPH in FWs with nonspecific neck/shoulder MP.

Concerning LT strength as influencing factor for PPH, scapulothoracic muscle imbalances could be the cause of impaired biomechanics, postural adaptations, and neck/shoulder pain.^[47,48]^ These imbalances may occur when the UT becomes tight and the LT becomes weak.^[49,50]^ Conversely, LT weakness could result in UT overload because of poor scapular mechanics (e.g., increasing scapular elevation and decreasing scapular upward rotation and posterior tilting)^[47,48]^ and weakly synergistic acceleration of UT overactivation (e.g., involving the serratus anterior and LT).^[51]^ LT strength was significantly different between the ipsilateral (mean ± standard deviation (SD): 21.8 ± 10.0 N) and contralateral sides (mean ± SD: 25.7 ± 11.5 N) in individuals with unilateral neck and shoulder pain.^[50]^ In the current study, LT strength in male FWs with PPH was 31.61% ± 15.58% normalized by body weight. Before LT strength was divided by body weight, LT strength was 23.50 N, which was similar to the results of a previous study involving individuals with neck/shoulder pain.^[50]^ However, Shahidi et al^[42]^ investigated physical influencing factors of chronic neck pain. They found that LT strength was not an influencing factor, using a multivariate prediction model that involved cervical active ROM, cervical muscle strength and endurance, and scapular muscle strength. Although this explanation is limited by the cross-sectional study design, LT could be linked to PPH of the UT and could potentially be weaker in terms of PPH of the UT. The process may function in an inverse manner.

Cervical mobility as a influencing factor for neck/shoulder pain has been suggested in prospective studies of other populations, but the results have been conflicting. Reduced cervical flexion mobility was more likely to cause neck/shoulder pain in laundry workers (risk ratio: 3.1; 95% CI = 1.2–8.3)^[52]^ and increased cervical flexion–extension mobility was protective against neck/shoulder pain in office workers (hazard ratio: 0.97; 95% CI: 0.94–0.99).^[53]^ With respect to dominant painful ipsilateral cervical rotation ROM as a influencing factor for PPH, cervical rotation ROM is related to pain intensity in patients with chronic neck/shoulder pain.^[54,55]^ Moreover, patients with nonspecific neck/shoulder pain show less cervical rotation ROM, compared to asymptomatic controls.^[56,57]^ Reduced extensibility of upper quadrant neural structures evaluated by the median nerve tension test has been related to decreasing UT length.^[58]^ Furthermore, the presence of PPH in the UT was associated with cervical intervertebral joint dysfunctions.^[59]^ Although interpretations are restricted by the cross-sectional study design, dominant painful side ipsilateral cervical rotation ROM could be linked to PPH of the UT and could potentially cause shortness involving PPH of the UT, or the process could function in an inverse manner. UT length affects ipsilateral cervical rotation ROM and contralateral cervical side-bending ROM because of the muscle attachment locations.^[60]^ Thus, tenderness or PPH of the UT could affect the restriction of cervical ipsilateral rotation ROM. Conversely, reduced UT length could affect PPH by scapular dyskinesis (e.g., scapular elevation during arm lifting).^[60]^ UT shortness and scapular dyskinesis could generate reduced activity of the serratus anterior and/or LT, as well as enhanced activity of the UT, resulting in UT overactivation.^[61,62]^

The psychological domain (depressed mood), as measured using the BDI, was not significantly different between FWs with PPH of the UT and FWs without PPH of the UT in both men (*P* = .988) and women (*P* = .666). This might have been due to limited statistical power resulting from the small sample size in this study. Psychological depressed mood was reportedly associated with an enhanced risk for neck pain in office workers (OR = 3.36; 95% CI = 1.10–10.31; *P* = .03)^[42]^ and others.^[63,64]^ Although it is difficult to directly compare our findings with the results of previous studies, a possible reason for exclusion of the psychological domain from the variable selection process was that the psychological domain could more weakly influence PPH among workers with repetitive and high physical load tasks, compared to white-collar office workers. Furthermore, physical domains of cervical and scapular posture were not significantly different between FWs with PPH of the UT and FWs without PPH of the UT in both men and women. Forward head posture^[65,66]^ and scapular posture^[19]^ have been previously associated with neck/shoulder pain. Because cervical and scapular posture are static characteristics, dynamic physical domains could more strongly influence PPH among workers with repetitive and high physical load tasks, rather than static physical domains.

The main limitation of the present study was its small sample size measured in 1 theme park. Furthermore, this study involved a relatively homogeneous sample of FWs. Future studies are necessary to determine whether the influencing factors identified in this study can be generalized to other demographic populations and professions. In addition, the design of present study was cross-sectional study. It is difficult to determine the direction of association between the variables in a cross-sectional study design. Thus, longitudinal study is necessary to determine causality of PPH in a future study. Furthermore, we measured depression in only the psychological domain. It is necessary to confirm the relationship among various psychological domains (anxiety, embarrassment, and job stress), social domains (race, culture, years of work experience) and musculoskeletal pain in future studies. Finally, future studies should also determine whether improvements in LT strength and cervical rotation ROM are effective for reducing PPH among individuals with neck/shoulder MP.

## 5. Conclusion

The present study investigated physical and psychological influencing factors of PPH of the UT in each sex among FWs with nonspecific neck/shoulder MP. LT strength and dominant painful ipsilateral cervical rotation ROM were influencing factors of PPH of the UT in men and women in adjusted multivariate analyses. Improvements in LT strength and dominant painful ipsilateral cervical rotation ROM may be protective against PPH in FWs with nonspecific neck/shoulder MP.

## Acknowledgments

We would like to thank all of the participants for their time and commitment to the present study.

## Authorship contributions

UJ Hwang: Conceptualization, project administration, writing-original draft, data curation, investigation, methodology, formal analysis, methodology.

OY Kwon: Conceptualization, funding acquisition, project administration, writing-original draft.

## Supplementary Material



## Footnotes

* *P* < .05.

* *P* < .05.

* *P* < .05.

* *P* < .05.
